# Genetic Profile, Environmental Exposure, and Their Interaction in Parkinson's Disease

**DOI:** 10.1155/2016/6465793

**Published:** 2016-01-31

**Authors:** Letizia Polito, Antonio Greco, Davide Seripa

**Affiliations:** ^1^Golgi Cenci Foundation, Abbiategrasso, 20081 Milan, Italy; ^2^Geriatric Unit and Gerontology-Geriatrics Research Laboratory, Department of Medical Sciences, IRCCS Casa Sollievo della Sofferenza, San Giovanni Rotondo, 71013 Foggia, Italy

## Abstract

The discovery of causative mutations for Parkinson's disease (PD) as well as their functional characterization in cellular and animal models has provided crucial insight into the pathogenesis of this disorder. Today, we know that PD pathogenesis involves multiple related processes including mitochondrial dysfunction, oxidative and nitrative stress, microglial activation and inflammation, and aggregation of *α*-synuclein and impaired autophagy. However, with the exception of a few families with Mendelian inheritance, the cause of PD in most individuals is yet unknown and the identified genetic susceptibility factors have only small effect size. Epidemiologic studies have found increased risk of PD associated with exposure to environmental toxicants such as pesticides, organic solvents, metals, and air pollutants, while reduced risk of PD associated with smoking cigarettes and coffee consumption. The role of environmental exposure, as well as the contribution of single genetic risk factors, is still controversial. In most of PD cases, disease onset is probably triggered by a complex interplay of many genetic and nongenetic factors, each of which conveys a minor increase in the risk of disease. This review summarizes the current knowledge on causal mutation for PD, susceptibility factors increasing disease risk, and the genetic factors that modify the impact of environmental exposure.

## 1. Introduction

Nineteen years ago, the discovery of the first genetic mutation responsible for Parkinson's disease (PD), p.A53T in the *α*-synuclein (*SNCA*) gene [1], provided the initial insights into the molecular genetics of PD. This finding was followed by data showing that *α*-synuclein is the major component of Lewy bodies (LB), a hallmark lesion in PD and other *α*-synucleinopathies [2]. Since then, an intensive search for other genetic causes for PD was launched and other mutated genes were reported to cause autosomal dominant or recessive forms of PD. Although monogenic forms are rare and altogether represent less than 10% of all PD cases [3], their functional characterization in cellular and animal models provided valuable insights into PD etiologic mechanisms. Recent advances of fundamental processes involved in neuronal death, particularly in the substantia nigra pars compacta, converge on abnormal endocytosis and endosome trafficking [4]. Starting from these dysfunctional mechanisms, multiple related processes, including mitochondrial dysfunction, oxidative and nitrosative stress, microglial activation and inflammation, and aggregation of *α*-synuclein and impaired autophagy, derive [5]. Besides rare causative mutations, several genetic susceptibility loci were discovered but with small to modest effect sizes [6].

Exploring the contribution of environmental exposure markedly advanced our understanding of the mechanisms involved in the development of PD. Initial evidence came from findings that subjects exposed to 1-methyl-4-phenyl-1,2,3,6-tetrahydropyridine (MPTP) developed PD-like symptoms [7]. Since then, environmental exposure to pesticides [8, 9], polychlorinated biphenyls [10], organic solvents [11], metals [12], and air pollutants [13] has been proposed to increase risk for PD. However, results concerning the contribution of environmental factors in PD are still inconsistent.

Altogether, although genes are likely to play a role, the vast majority of PD cases cannot be ascribed exclusively to genetic factors. PD is probably caused by a complex interplay of many genetic variants interacting with many nongenetic risk factors.

Here, we briefly review research on the genetic and environmental causes of PD. We also summarize evidence on gene-environment interplay in the development of PD with an emphasis on positive findings. Anyway, negative studies will be cited. Furthermore, positive results from human association studies should be interpreted with caution as most of these studies are based on a relatively small number of exposed subjects. Certainly, more large-scale human association studies aimed at identifying gene-environment interactions in the development of PD may prove to be fruitful.

## 2. Monogenic Forms of PD

Mutations in two genes (*SNCA* and* LRRK2*) cause autosomal dominant forms of PD with peculiar features. Mutations in the* SNCA* gene are rare and highly penetrant and generally cause early onset autosomal dominant inherited forms of PD [3]. Besides the above mentioned p.A53T mutation in the* SNCA* gene, other point mutations in the same gene (p.A30P [14], p.E46K [15], p.H50Q [16], and p.G51D [17]), as well as duplications and triplications of the locus containing the* SNCA* gene [18], were identified to cause PD. Brain pathology in* SNCA* mutation carriers is characterized by diffuse LB pathology and Lewy neurites (LNs) [19]. Clinical features of* SNCA* mutation carriers range from classical symptoms (bradykinesia, muscle rigidity, resting tremor, and postural instability), and good response to levodopa therapy, to more atypical phenotypes resembling other synucleinopathies (Lewy body dementia or multiple system atrophy) [20].

Mutations in the leucine-rich repeat kinase 2 (*LRRK2*) gene are less rare and have incomplete and age-dependent penetrance. They generally cause late onset autosomal dominant inherited forms of PD. Although almost 80 gene variants have been identified in this large gene in PD patients, only seven of these (p.N1437H, p.R1441C, p.R1441G, p.R1441H, p.Y1699C, p.G2019S, and p.I2020T) can be considered as definitely disease causing mutations [21]. LB pathology is also the dominant pathology in most cases of* LRRK2*-related PD along with, more rarely, tau or TDP-43 pathology [22]. However, in some cases, LB pathology is not observed. Clinical features resemble classical motor symptoms and good response to levodopa therapy.* LRRK2* p.G2019S is the most common known cause of autosomal dominant PD, accounting for 1–40% of sporadic or dominantly inherited PD, depending on the population examined. The worldwide frequency of* LRRK2* p.G2019S was 1% of patients with sporadic PD [23]. The highest prevalence rates were registered for Ashkenazi Jewish [24] and North African Arab [25] populations, where* LRRK2* p.G2019S accounts for approximately 20% and 40% of PD cases, respectively.

Recently, mutations in three novel genes, that is, the vacuolar protein sorting 35 homolog (*VPS35*), eukaryotic translation initiation factor 4 gamma 1 (*EIF4G1*), and dnaJ homolog subfamily C member 13 (*DNAJC13*), were proposed to cause late onset autosomal dominant inheritance and need further replication to be confirmed [26–28].

Loss-of-function mutations in Parkin (*PARK2*,* PRKN*), PTEN-induced putative kinase 1 (*PINK1),* and Daisuke-Junko-1 (*DJ-1*) cause rare forms of autosomal recessive Parkinsonism with early onset and slow progression [29–31]. Recessively inherited forms of atypical Parkinsonism with juvenile onset are caused by mutations in the ATPase type 13A2 (*ATP13A2*), phospholipase A2 group VI (*PLA2G6*), and F-box only protein 7 (*FBXO7*) genes [32–34].

## 3. Genetic Variants Associated with PD

It has been estimated that about 90% of PD patients have no family history [35]. With the exception of a few families with Mendelian inheritance, PD etiology is most likely caused by the combination of several genetic and environmental factors [36]. Candidate gene association studies as well as genome-wide association studies (GWAS) have identified polymorphisms in a number of genes that were significantly related to the development of PD. Some of these were consistently replicated while, for the others, the true significance remains to be examined [6].

Candidate gene association studies focused on selected genes that were genetically, clinically, or functionally related to PD. Great effort has been spent in the last 20–30 years in this research field. This kind of approach was in many cases unsuccessful with some notable exceptions [20]. Several studies explored disease risk associated with allelic variants of genes already linked to monogenic PD or to other neurological diseases. For example, p.G2385R in the* LRRK2* gene is common among Chinese and Japanese populations and approximately doubles the risk for PD [37], while the REP1 microsatellite marker of the* SNCA* promoter region was consistently associated with a 1.4-fold increased risk of PD [38]. Additionally, the H1 haplotype of microtubule-associated protein tau (*MAPT*) gene has been identified as a risk factor for idiopathic PD [39]. Clinical observations led to the identifications that some gene variants, known to cause other diseases, were associated with higher risk for PD. Some examples are variants in the glucocerebrosidase (*GBA*) [40], the sphingomyelin phosphodiesterase 1 (*SMPD1*) [41], and the GTP cyclohydrolase 1 (*GCH1*) [42] genes, responsible for Gaucher's disease, Niemann-Pick A disease, and dopa-responsive dystonia (DRD), respectively.

Several GWAS have been performed to investigate the influence of common genetic variations in PD. The first GWAS confirmed the causal genes* SNCA*,* LRKK2,* and* MAPT* as risk genes also for idiopathic PD [43, 44]. Subsequent GWAS and meta-analyses revealed additional risk genes. Recently, meta-analysis pooling data from 15 PD GWAS, including 13,708 patients and 95,282 control individuals, identified 28 independent single nucleotide polymorphisms (SNPs) as susceptibility variants for PD across 24 different loci [6]. Although the effect of each individual locus was small, risk profile analysis showed substantial cumulative susceptibility in a comparison of the highest and lowest quintiles of genetic risk [6], suggesting that the risk for PD increases with the number of susceptibility alleles carried by a single subject.

## 4. Environment Factors Related to PD

Exploring the contribution of environmental exposure markedly advanced our understanding of the mechanisms involved in the development of PD. Since the initial evidence regarding 1-methyl-4-phenyl-1,2,3,6-tetrahydropyridine (MPTP) [7], a number of studies have reported the association between exposure toxicants and increasing risk of developing PD. Among these, pesticides (e.g., rotenone, paraquat, dichlorodiphenyltrichloroethane, dieldrin, and organophosphates) have been largely studied [8, 9]. A recent meta-analysis of 46 studies from around the world found a summary risk ratio of 1.62 (95% CI [1.40–1.88]) for pesticide exposure (ever versus never) [9]. In particular, in a recent case-control study examining the risk of developing PD based on exposure to 31 specific pesticides, 2 were found to increase risk: paraquat (OR = 2.5; 95% CI, 1.4–4.7) and rotenone (OR = 2.5; 95% CI, 1.3–4.7) [45]. Besides pesticides, other toxicants were proposed to increase risk for PD, such as polychlorinated biphenyls [10], solvents [11], metals [12], and air pollutants [13].

In contrast, possible protective factors include cigarette smoking and coffee and tea consumption [46]. Risk in ever-smokers is half of that in never-smokers, and there is a clear dose-response relationship. Caffeine and coffee consumption were also consistently associated with reduced risk of PD; the magnitude of the reduced risk is similar to that of smoking, and a dose-response relationship is evident [47].

Overall, results of epidemiological studies, concerning the contribution of environmental toxicants in PD, are sometimes inconsistent. Identifying subpopulations at different genetic-based risk is one way to improve the study design. In this regard, the next two sections will be focused on the relevance of genetic polymorphisms in toxicokinetics and toxicodynamics in PD. Several interesting findings will be reported although sometimes not replicated, as mentioned in the relevant section within the paper. Positive findings related to the joint gene-environment contribution to PD susceptibility are also summarized in Table 1.

## 5. The Role of Genetic Variants in the Kinetics of Environmental Factors

Recently, epidemiologic studies have begun to consider the joint effects of toxicant exposure and polymorphisms in genes that affect the toxicants' absorption, metabolism, and excretion.

### 5.1. Absorption

P-glycoprotein (P-gp) is an efflux transporter encoded by the* ABCB1* (also known as* MDR1*) gene that protects the brain against neurotoxicants [66]. Certain* ABCB1* genetic variants, known to alter the function of this transporter, have been suggested to influence the risk to develop PD in conjunction with exposure to toxicants [48–50, 67]. A case-control study, in 599 European PD patients and controls, detected no relevant association between three* ABCB1* variants and PD, while it found that the distribution of c.3435C>T differed significantly between PD patients exposed to pesticides compared to those nonexposed (OR = 4.74, 95% CI [1.01–22.31]) [48]. Another case-control study, among 207 PD cases and 482 matched controls, addressed the association between PD and 2 polymorphisms in* ABCB1* (c.2677G>[A/T], c.3435C>T), as well as the interaction between* ABCB1* and pesticides. Participants were classified as never users, user for gardening, and professional users of pesticides. This study found that* ABCB1* polymorphisms were not associated with PD. Among PD cases only, an association between carrying 2 variant c.2677G>[A,T] alleles and organochlorine exposure was found (OR = 5.4, 95% CI [1.1–27.5]) [49]. More recently, another study lent support to previous findings. In a population-based case-control study, including 350 cases and 724 controls, homozygote carriers of* ABCB1* c.2677G>[A/T] or/and c.3435C>T risk alleles, exposed specifically to organophosphorus pesticides, had from 2 to 3.7 times higher risk to develop PD versus noncarriers (OR = 2.1, 95% CI [1.3–3.2] for homozygotes of 1 risk allele; OR = 3.7, 95% CI [2.0−7.0] for homozygotes of both risk alleles) [50]. In contrast to all these reports mainly relating to participants of European ancestry, a Japanese hospital-based case-control study found no interaction between pesticide exposure and* ABCB1* rs1045642 [68]. Reason for this inconsistency could be that, unlike previous cited studies, authors examined interactions for rs1045642 using a dominant genetic model or might be explained by ethnic differences.

### 5.2. Metabolism

Paraoxonases and cytochromes P450 constitute two major classes of xenobiotic-metabolizing enzymes involved in the detoxification of pesticide chemicals.

One study investigated a functional polymorphism of the Paraoxonase I (*PON1*) gene (c.260T>A, p.L55M) on 351 incident PD cases and 363 controls taking into account residential exposure to organophosphates (OP). This study found that carriers of the “slower” metabolizer genotype (AA), exposed to OP (diazinon, chlorpyrifos), exhibited a greater than 2-fold increase in PD risk compared with persons who had the wild-type or heterozygous genotype and no exposure [51]. More recently, the same group extended its previous finding showing that several* PON1* variants may act together to modify PD risk for ambient OP pesticide exposure. Carriers of both* PON1* p.L55M and* PON1* p.Q192R slow metabolizer variants were more susceptible to pesticide exposure (e.g., for chlorpyrifos-exposed carriers of the MM-QQ diplotypes, OR = 3.28, 95% CI [1.02–10.58]) compared to those unexposed with a LL-RR diplotype [69]. Recently, a population-based case-control study suggested that household pesticide use increases the odds of developing PD especially for products that contain OP. Furthermore, exposed participants' carriers of* PON1* p.Q192R QQ variant were at higher risk than noncarriers who were rarely exposed or unexposed (OR = 2.62, 95% CI [1.4–4.8]) [52]. Lack of interaction was also reported in studies of the early 2000s [70, 71].

CYP2D6 is one of the CYP superfamilies of enzymes, which metabolizes several xenobiotics in the liver, including OP pesticides, the herbicide atrazine, and 1-methyl-4-phenyl-1,2,3,6- tetrahydropyridine (MPTP). The activity of CYP2D6 is largely determined by genetic variability and common sequence variants exist in human populations that lead to poor metabolizer (PM) phenotypes [72]. These variants have been extensively studied as genetic risk factors for PD with inconsistent results. Two independent studies regarded gene-environment interactions and suggested that CYP2D6 poor metabolizers (PM), who were exposed to pesticides, exhibited an increased risk for PD both compared with unexposed subjects and pesticide-exposed CYP2D6 extensive metabolizers (EMs) [53, 54]. More recently, another study confirmed these findings [73]. Negative results were also reported [55].

Caffeine that was proposed to be a protective factor for PD is primarily metabolized by cytochrome P450 1A2 (CYP1A2). A study, investigating three* CYP1A2* polymorphisms, found that the coffee-PD association was the strongest among subjects homozygous for either variant allele c.-164A>C (*p* for interaction = 0.05) or c.1545T>C (*p* for interaction = 0.04) (i.e., slow metabolizers of caffeine) [56].

### 5.3. Excretion

The role of glutathione S-transferases M1 (GSTM1), T1 (GSTT1), and P1 (GSTP1), involved in the detoxification of many xenobiotics, was explored. A recent study found that paraquat exposure, a herbicide structurally similar to MPP+, had little association with PD in individuals carrying two active copies of the* GSTT1* gene (OR = 1.5, 95% CI [0.6–3.6]), while markedly increasing PD risk in those with homozygous* GSTT1* gene deletions (OR = 11.1, 95% CI [3.0–44.6]) [74]. Another study proposed that herbicide exposure may be an effect modifier of the relation between* GSTP1* polymorphisms and age at onset in familial PD. Exposure to herbicides was classified as absent, residential, or occupational exposure. Seven SNPs in the* GSTP1* gene were genotyped. The strongest result regarded the rs762803–rs1799811 haplotype that was associated with an approximately 8-year-earlier onset in the occupationally exposed group and a 2.8-year-later onset in the nonexposed group [57]. Another evidence regarded a case-control study of 959 prevalent cases of Parkinsonism (767 with PD) and 1,989 controls across five European centers, where the average annual intensity of exposure to solvents, pesticides, and metals was estimated. This study found possible interaction effects between* GSTM1* null genotype and solvent exposure in PD patients only.* GSTM1* null subjects heavily exposed to solvents appeared to be at increased risk of PD [55].

Cigarette smoking is thought to reduce risk of PD, and emerging evidence suggests that genetic factors may modulate smoking's effect. One study, with a case-only design in four hundred PD cases, assessed interactions between GST gene polymorphisms and smoking in relation to PD and found that* GSTP1* ^*∗*^ *C* haplotypes were overrepresented among PD cases who ever smoked (OR = 2.00, 95% CI [1.11–3.60]). Noteworthy, the statistical significance of the interaction between smoking and the* GSTP1* p.A114V VV carrier status increased with increasing smoking dose (*p* = 0.02 for trend). These data suggest that one or more GSTP1 polymorphisms may interact with cigarette smoking to influence the risk for PD [58].

## 6. The Role of Genetic Variants in the Dynamics of Environmental Factors

The dopamine transporter gene (*SLC6A3*) is a candidate gene for PD on the basis of its critical role in dopaminergic neurotransmission. A couple of studies suggested that* SLC6A3* genetic variability and pesticide exposure interact to increase PD risk [59, 60]. The first study explored 5′ A clade and 3′ VNTR 9-repeat allele risk variants in the* SLC6A3* and occupational pesticide exposure. This study found that, among pesticide-exposed subjects, the odds ratio for having two or more risk alleles was 5.66 (95% CI: 1.73–18.53), while in nonexposed subjects it was 1.17 (0.62–2.23) [59]. The second study explored again the 5′ and 3′ regions of* SLC6A3* and determined residential exposure to agricultural maneb and paraquat applications. This study found that high exposure to paraquat and maneb in carriers of one susceptibility allele was associated with a 3-fold increased PD risk (OR = 2.99, 95% CI [0.88–10.2]), while it was associated with a 4-fold increased risk in carriers of two or more alleles (OR = 4.53, 95% CI [1.70–12.1]) [60]. In line with epidemiological findings, a molecular study reported that paraquat, when converted to the monovalent cation PQ(+) by either a reducing agent or NADPH oxidase on microglia, is transported by DAT and is accumulated in dopaminergic neurons, where it induces oxidative stress and cytotoxicity [75].

Another interesting target to study is monoamine oxidase B (MAO-B) since this enzyme breaks down dopamine. MAO-B inhibitors are used to treat the symptoms of PD since they prolong the action of dopamine in the brain. The A/G polymorphism in intron 13 of the* MAO-B* gene has been associated with variability of the MAO-B enzyme activity. In a population-based case-control study, a reversal of the association of cigarette smoking with PD in relation to this polymorphism was found. A reduced PD risk related to pack-years of smoking was detected for persons with the G allele, whereas an opposite effect was found among persons with the A allele [61]. Another study, on 186 incident idiopathic PD cases and 296 matched controls, confirmed this result only in men. Indeed, gender-specific interactions between smoking and genetic polymorphisms of* MAO-B* intron 13 A/G polymorphism were found to influence PD risk (OR = 0.27, 95% CI [0.13–0.58] for ever-smokers versus never-smokers in men (genotype G), and OR = 1.26, 95% CI [0.60–2.63] for men of genotype A (interaction *χ* ^2^ = 8.14, *p* = 0.004)). No association was found for women [76].

Several case-control studies and genome-wide association studies have examined the relationships between single nucleotide polymorphisms (SNPs) in the SNCA gene and Parkinson's disease (PD) and have provided inconsistent results. Evidence for biological interactions between* SNCA* SNPs rs356219 and rs356220 and smoking that affect sporadic PD was presented [77].

ATP13A2 belongs to the P-type superfamily of ATPases that transport inorganic cations and other substrates across cell membranes. As already mentioned, mutations in the ATPase type 13A2 (*ATP13A2*) gene are cause of recessively inherited forms of atypical Parkinsonism. Recently, it has been proposed that* ATP13A2* variations may be a risk marker for neurotoxic effects of manganese (Mn) in humans. Mn intoxication can lead to a disorder known as manganism characterized by severe neurological deficits that often resemble the involuntary extrapyramidal symptoms associated with Parkinson's disease and may evolve to more Parkinson-like syndrome [78]. A study, settled in Val Camonica (Italy), a geographic area with higher prevalence of individuals affected by Parkinsonism, probably related to increased exposure to Mn in the air, soil, and water, examined individual susceptibility for Mn neurotoxicity. It examined whether polymorphism in genes regulating Mn metabolism and toxicity could modify neurophysiological effects of Mn exposure. It found that* ATP13A2* polymorphisms rs4920608 and rs2871776 significantly modified the effects of Mn exposure on impaired motor coordination in the elderly (*p* for interaction = 0.029, *p* = 0.041, resp.) [79].

Oxidative and nitrosative stress plays an important role in the degeneration of dopaminergic neurons in PD. Key antioxidant enzymes such as NAD(P)H:quinone oxidoreductase (NQO1) and manganese superoxide dismutase (MnSOD) are polymorphic. Individual vulnerability to oxidative stress due to genotypic polymorphisms and exposure to environmental xenobiotics has been considered to promote the development of PD. In a study from southwestern region of Taiwan, it was investigated whether functional variants of* MnSOD* and* NQO1* genes interacted with occupational pesticide exposure to increase PD risk. A total of 153 patients with idiopathic PD and 155 healthy controls were genotyped for* MnSOD* (rs4880) and* NQO1* (rs1800566) genetic variants. This study found significant differences in frequencies of both genotypes of* MnSOD* and* NQO1* polymorphisms between PD patients and the control subjects only among subjects who had been exposed to pesticide (OR = 2.49, 95% CI [1.18–5.26] for* MnSOD* C allele; OR = 2.42, 95% CI [1.16–4.76] for* NQO1* T allele). Moreover, among those exposed to pesticide, the combined* MnSOD* and* NQO1* variant genotype was significantly associated with a 4.09-fold increased risk of PD (OR = 4.09, 95% CI [1.34–10.64]) [62].

Nitric oxide synthase (NOS) may create excess nitric oxide that contributes to neurodegeneration in PD. A study, examining gene-environment interactions involving both pesticides and protective factors (cigarette smoking, caffeine, and nonsteroidal anti-inflammatory drugs), found significant interactions between pesticides exposure and the* NOS1* SNPs rs12829185, rs1047735, and rs2682826 in determining the risk of PD (range of *p* = 0.012–0.034). Interactions between* NOS2A* SNPs rs231480, rs2248814, and rs1060826 and smoking were also found (range of *p* = 0.013–0.024) [63]. A recent study, in 357 incident PD cases and 495 population controls, investigated 8* NOS* SNPs and interactions with both household and ambient agricultural OP pesticide exposures. The OR for frequent household OP use combined with the presence of* NOS1* rs2682826 C/T CT+TT genotype was 2.84 (95% CI [1.49, 5.40], interaction *p* value 0.04), while combined with* NOS1* rs3741480 T/C CT+CC genotype it was 1.90 (95% CI [1.06–3.41], interaction *p* value 0.02). Similar results were seen for ambient OP exposure (*NOS1* rs1047735 C/T OR = 5.42, 95% CI [2.54–11.52], interaction *p* value 0.04;* NOS1* rs816353 G/T OR = 4.24, 95% CI [2.30–7.83], interaction *p* value 0.03;* NOS1* rs3741480 T/C OR = 3.78, 95% CI [2.04–6.99], interaction *p* value 0.01) [80].

Epidemiological, clinical, and animal studies provided a comprehensive picture of the anti-Parkinsonian potential of caffeine. A recent genome-wide association and interaction study (GWAIS) identified* GRIN2A*, which encodes an NMDA-glutamate-receptor subunit involved in brain's excitatory neurotransmission, as a PD genetic modifier in inverse association with caffeine intake (*p*_interaction_ = 3 × 10^−5^) [64]. This result was questioned by another group that performed a reanalysis of the same data by examining the association between coffee and rs4998386 separately in cases and controls. This group found a strong positive association in controls between rs4998386-T and heavy coffee drinking (OR = 1.48, 95% CI [1.23–1.78]). On the contrary, among PD cases, heavy coffee drinking tended to be less frequent in carriers of the rs4998386-T allele, but this association was not statistically significant (OR = 0.82, 95% CI [0.65–1.03]). Therefore, it appeared that the interaction between rs4998386 and coffee consumption was in part explained by a positive association between the rs4998386-T allele and coffee consumption among controls, but not among PD cases [81]. An independent study replicated the reported association of a single nucleotide polymorphism,* GRIN2A* rs4998386, and its interaction with caffeine intake with PD in patient-control study in an ethnically homogenous population in southeastern Sweden in 193 sporadic PD patients and 377 controls. There was also a strong significance in joint effects of gene and caffeine on PD risk (TC heavy caffeine versus CC light caffeine: OR = 0.38, 95% CI [0.20–0.70], *p* = 0.002) and gene-caffeine interaction (OR = 0.998, 95% CI [0.991–0.999], *p* < 0.001) [65].

## 7. Conclusion

The discovery of causative mutations for PD as well as their functional characterization in cellular and animal models has provided crucial insight into the pathogenesis of this disorder. Candidate gene association studies as well as genome-wide association studies (GWAS) have identified polymorphisms in a number of genes that significantly correlate with the development of PD. Some of these were consistently replicated while for others the true significance remains to be examined. Recent advances have revealed that certain interactions modify the risk of PD. However, few studies have examined gene-environment interactions, probably because of some limitations such as the need of large sample size and difficulties in estimating exposures, particularly for toxicants. In the future, it will be crucial to consider genetic and environmental exposure cooccurrence for PD prevention and personalized medicine to treat this disease.

## Figures and Tables

**Table 1:** Environmental genetic significant interactions in Parkinson's disease.

Exposure	Gene	Risk variant^&^	Design for interaction	PD	CT	Interaction (*p*)	Joint effect	REF
							OR [95% CI] or (SE)	
Pesticides	*ABCB1*	rs1045642 C>T, p.Ile1145Ile	Case-only	415	—	—	4.74 [1.01–22.31]	[48]
Organochlorines	*ABCB1*	rs2032582 G>[A,T], p.Ser893Ala/Thr	Case-only	207	—	—	5.4 [1.1–27.5]^*∗*^	[49]
Organophosphorus	*ABCB1*	rs1045642 C>T, p.Ile1145Ile rs2032582 G>[A,T], p.Ser893Ala/Thr	Case-control	350	724	NA	1 allele 2.1 [1.3–3.2]^*∗*^ Both alleles 3.7 [2.0–7.0]^*∗*^	[50]
Diazinon Chlorpyrifos	*PON1*	rs854560 T>A, p.Leu55Met (SM)	Case-control	351	363	NA	2.2 [1.1–4.5] 2.6 [1.3–5.4]	[51]
Organophosphates	*PON1*	rs854560 T>A, p.Leu55Met (SM)	Case-control	357	807	NA	2.62 [1.4–4.8]	[52]
Pesticides	*CYP2D6*	rs3892097 G>A, null allele (PM)	Case-control	190	419	0.02	4.74 [1.29–17.45]	[53]
Pesticides	*CYP2D6*	rs3892097 G>A, null allele (PM)	Case-control	393	389	0.05	8.41 [1.01–69.76]	[54]
Caffeine	*CYP1A2*	rs762551 C>A rs2470890 C>T, p.Asn516Asn	Case-control	925	1249	0.05 0.04	0.33 [0.16–0.68]^$^ 0.43 [0.27–0.69]^$^	[55]
Paraquat	*GSTT1*	Null allele	Case-control	87	343	0.027	11.1 [3.0–44.6]	[56]
Solvents	*GSTM1*	Null allele	Case-only	959	—	—	2.34 [1.08–4.62]	[57]
Smoking	*GSTP1*	GSTP1^*∗*^C haplotype	Case-only	400	—	—	2 [1.11–3.60]	[58]
Pesticides	*SLC6A3*	5′ A clade and 3′ VNTR 9-repeats	Case-control	178 men	239 men	0.02	5.66 [1.73–18.53]^*∗*^	[59]
Paraquat, maneb	*SLC6A3*	5′ A clade and 3′ VNTR 9-repeats	Case-control	324	334	<0.001	4.53 [1.70–12.09]	[60]
Smoking	*MAO-B*	rs1799836 A>G	Case-control	82	118	NA	0.24 [0.10–0.55]^$^	[61]
Pesticides	*MnSOD* *NQO1*	rs4880 T>C p.Val16Ala rs1800566 C>T p.Pro153Ser	Case-control	153	155	<0.001	2.49 [1.18–5.26] 2.42 [1.16–4.76]	[62]
Pesticides	*NOS1*	rs12829185 T>C rs10774910 T>C rs2682826 A>G	Case-control	156	174	0.034 0.026 0.028	3.12 [1.71–5.71]^$^ 4.15 [1.85–9.34]^$^ 3.52 [1.78–6.95]^$^	[63]
Smoking	*NOS2A*	rs2314810 G>C rs2248814 A>G rs1060826 T>C	Case-control	179	204	0.024 0.021 0.013	0.56 [0.34–0.92]^$^ 0.23 [0.09–0.59]^$^ 0.17 [0.06–0.49]^$^	[63]
Caffeine	*GRIN2A*	rs4998386 C>T	GWAIS + replications	2472	2848	3 × 10^−5^	0.41 (0.05)	[64]
Caffeine	*GRIN2A*	rs4998386 C>T	Case-control	193	377	<0.001	0.38 [0.20–0.70]	[65]

^&^ http://www.ncbi.nlm.nih.gov/projects/SNP/, ^*∗*^environmental exposure stratum, and ^$^risk allele stratum. PD, Parkinson's disease patients; CT, unaffected subjects; OR, odds ratio; CI, confidence interval; REF, reference article; NA, not available; SM, slow metabolizer; PM, poor metabolizer.
